# Simulated training model in a low cost for laparoscopic inguinal hernioplasty

**DOI:** 10.1590/ACB360108

**Published:** 2021-02-15

**Authors:** Luís Pires de Melo, Alexandra Mano Almeida, Edgar Marçal de Barros, Gleydson Cesar de Oliveira Borges

**Affiliations:** 1.MD. Santa Casa de Misericórdia de Fortaleza – Department of General Surgery – Fortaleza (Ceará), Brazil.; 2 MD. Santa Casa de Misericórdia de Fortaleza – Department of General Surgery – Fortaleza (Ceará), Brazil.; 3 PhD. Centro Universitário Christus – Postgraduate Program in Computer Science – Fortaleza (Ceará), Brazil.; 4 MD. Centro Universitário Christus – Professional Master`s Degree in Minimally Invasive Technology and Simulation in the Health field– Fortaleza (Ceará), Brazil.

**Keywords:** Inguinal Hernia, Laparoscopy, Simulation Training, Health Education, Three-Dimensional Printing

## Abstract

**Purpose:**

Develop a 3D model for the simulation of laparoscopic inguinal hernioplasty transabdominal preperitoneal (TAPP).

**Methods:**

This is an experimental study, 18 participants were selected, divided into three groups, experimental (GE) surgeons in training, control (GC) experienced surgeons and Shaw (GS) nonexperienced surgeons. The simulation in the 3D model was carried out in 6 sessions fulfilling the 5 stages. Opening the peritoneum with the creation of the preperitoneal space; identification of important structures; hernia identification and reduction; placement and fixation of the mesh in Cooper’s ligament and closure of the peritoneum.

**Results:**

In the 1st stage, the GE obtained an average of 1.25 ± 0.42 in the 1st session and 3.25 ± 0.62 in the 6th session (p = 0.05) and in the 5th stage 0.91 ± 0.29 in the first session. 1st session and 1.91 ± 0.29 in the 6th session (p = 0.001), with no significant difference between groups. The learning and skill curve in the SG represented 1.08 ± 0.29 1st and 3.50 ± 0.90 6th session (p = 0.001).

**Conclusions:**

The creation of a systematization of training in simulation applied to the three-dimensional model enabled gain in laparoscopic skills and underpinned its theoretical and practical foundations.

## Introduction

Inguinal hernia is a pathology described as a public health problem due to its prevalence in the adult population^1^. Its prevalence is estimated from 5 to 18% and the incidence reaches from 100 to 300 cases per 100,000 inhabitants yearly worldwide, being the inguinal hernioplasty the most commonly performed abdominal surgery^2^^–^ 4. Laparoscopic correction of inguinal hernias became popular in the 1990s when Ger first described this approach^5^. In the same decade, Maurice Arregui described the technique that combined the principles of the French school (STOPPA) with the new minimally invasive approach being named as transabdominal preperitoneal (TAPP)^6^.

The literature describes laparoscopy as an effective approach, reducing cost of painkillers, intraoperative and long-term complications such as seroma, numbness, and pain^7^^–^ 9. Even with the most serious complications being rare, they are intestinal perforations and large vessel lesions. Bladder lesion is more common, corresponding to 0.2%, being more frequent in patients with anterior suprapubic surgery^10^. Even with an effective approach the procedure cost became limiting, both in the procedure and in the learning curve^11^. But with the spread of knowledge and experience, it was possible to adapt and improve these factors, and with favorable patient performance due to lower metabolic response to trauma, early hospital discharge, rapid return to work, reduced days off due to disability, laparoscopy becomes an effective and efficient method that can provide better patient safety^11^^–^ 12.

The simulated training (ST) in laparoscopy demonstrates several benefits for not intervening in the patient, where both experienced surgeons and those in training can learn^13^. A cognitive and technical training curriculum is important for the optimization of surgeon skills. Thus, the availability of ST should be as early as possible to develop the necessary skills^14^. Therefore, this study aimed to develop a three-dimensional (3D) model for the simulation of TAPP laparoscopic inguinal hernioplasty and to evaluate its application in three groups of surgeons.

## Methods

This is an experimental study, developed and applied at the Laboratory of Surgical Skills (LSS) and the Laboratory of Technological Innovation (LTI) of Centro Universitário Unichristus. The sample was characterized by surgeons, divided into three groups, experimental group (EG) surgeons in training, control group (CG) experienced surgeons and Shaw group (SG) surgeons not experienced.

### Intervention description

The sessions were supervised by a research surgeon passively, without any intervention during the execution of the curriculum stages by the surgeons in training participating in the research. His function was strictly to measure the surgical time, record the session as described in the methodology and receive the forms. Participants had early access to the curriculum (Fig. 1), being able to read and clarify doubts in the execution steps and then watched the video exemplifying the execution of the curriculum in the HerniLap 3D model.

**Figur2 1:**
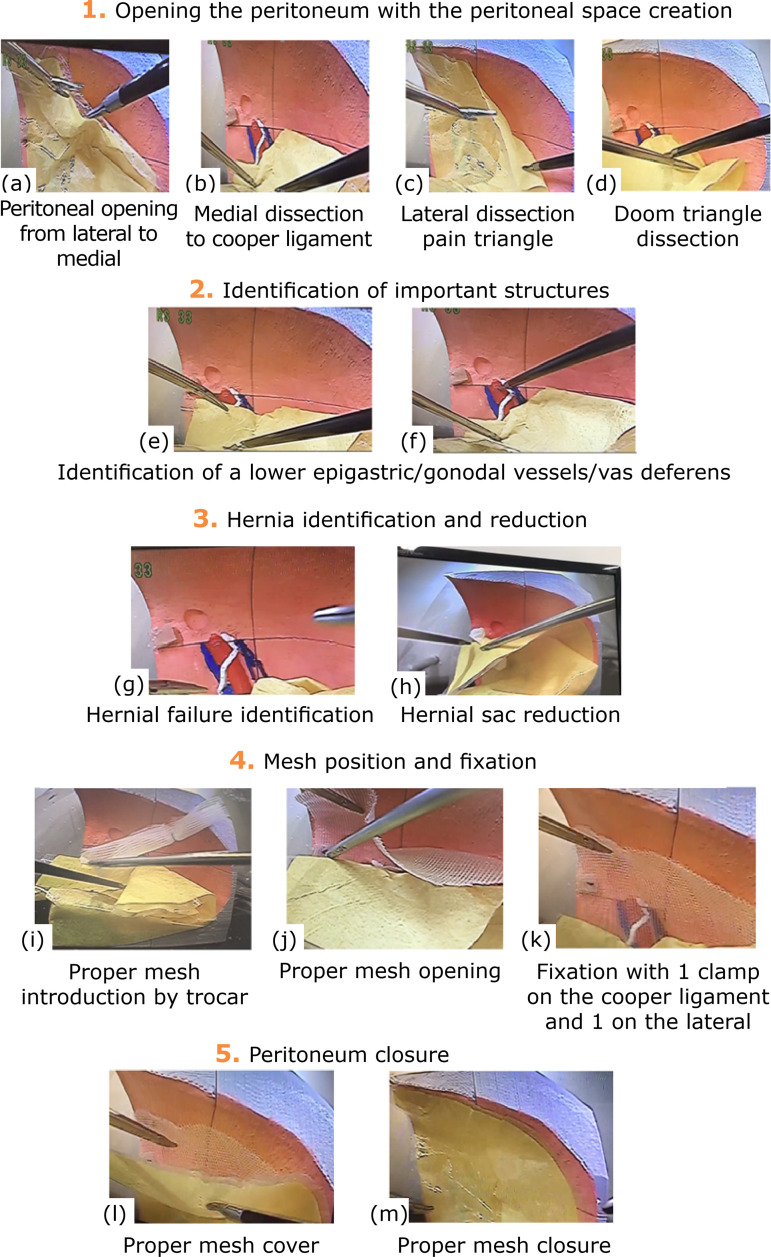
Systematic curriculum of surgical technical execution.

The ST developed in this study included five steps (Step 1: opening of the peritoneum to create the preperitoneal space; Step 2: identification of structures; Step 3: identification and reduction of the hernia; Step 4: mesh placing and fixing and Step 5: peritoneum closure, shown in Fig. 1). The training was conducted in 6 sessions. Surgeons’ experience with video games and limb dominance were evaluated.

### Eligibility criteria

For EG, surgeons who were in the second year of the General Surgery residency at a hospital at Fortaleza; be interested in the laparoscopic access route; have mastery over basic laparoscopic skills were included; for SG, surgeons who perform laparoscopy, but have no experience with inguinal hernia repair TAPP were included; and for CG, surgeons who have performed at least 60 TAPP inguinal hernioplasties were included.

The surgeons who didn’t complete the proposed training within six weeks; were not present during all training sessions; didn’t complete the forms and terms requested by the researcher; and didn’t have time compatibility were excluded.

### 3D model development and simulator adjustments

The first step was the collection of anatomical images in an atlas of human anatomy. The second step was the application of image patterns in the Blender software, version 2.79, used in the Windows 10 operating system. This anatomy was applied as a reference in a model file called Male Pelvis Section from TurboSquid. The third step involved the printing and suitability 3D prototype enabling the best way to realistic simulation.

The failure making in the 3D model simulating direct inguinal hernia was created by applying a sphere-shaped Boolean modifier to the pelvis model with the difference operation to remove from it the intersection part between the pelvis and the previously created sphere. In order to paint the model, two flat Condor brushes and gouache ink were used, using the standard colors, according to the anatomy books to characterize the structures present in the model.

**Figure 2:**
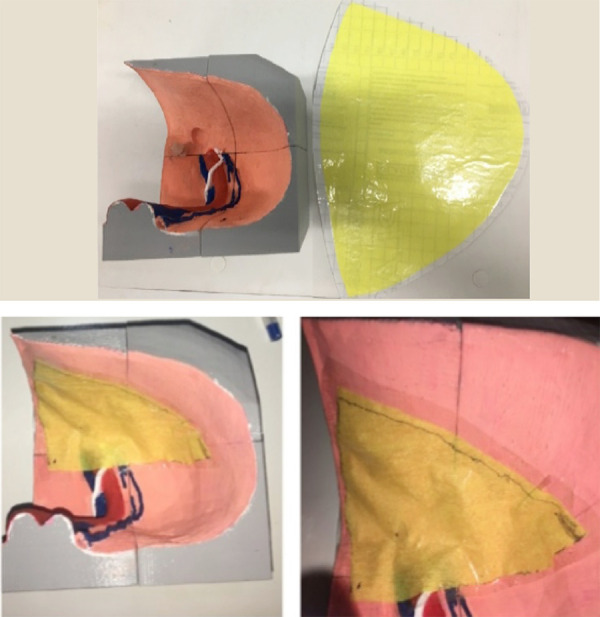
3D model with material apposition representing the peritoneum.

Next, a synthetic rubber was fixed very close to the hernial defect to simulate Cooper’s ligament allowing the stapler to tackers, fixing the mesh as the real one. The next step in the model construction consisted in making adhesive structures that simulated the pelvic peritoneum and thus allowed the opening time of the peritoneum in the technique execution. For this, masking tape and Contact adhesive paper were used, covering the entire internal model’s part, corresponding to the abdominal cavity and its peritoneum. Initially, there was a difficulty in adapting the material to the model’s surface as well as how much should be covered from the interior of the model (Fig. 2). After a critical analysis by the researcher, the best measures to coat the model were determined.

**Figure 3:**
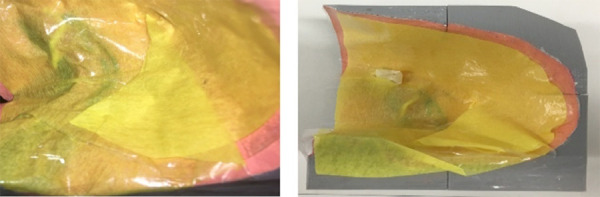
3D model with a glove representing the hernial sac.

In order to represent the hernial sac and its content to be reduced, a surgical time also important during the technique execution, it was used a latex glove inserted in the failure built in the model and adhered to the masking tape and Contact adhesive. In the model, it was possible to see the anatomical structures through transparency of masking tape and Contact adhesive, as well as in the real procedure. Once the model’s construction was finished, it was decided to call it HerniLap 3D (Fig. 3).

The last step was the placement of the model inside the simulator, located in the LSS. The best position, distance of the trocars and camera position were seen to represent the simulation as real as possible. After finishing the model making four similar prototypes were reproduced.

### Statistical analysis

Categorical data were expressed as absolute and percentage frequencies and compared by Fisher’s exact test or Pearson’s chi-square. The quantitative data were expressed as mean and standard deviation and compared among groups by the Kruskal–Wallis test and within the same group for the Friedman test measures both followed by the Bonferroni posttest. All analyzes were performed using 95% confidence in the Statistical Packing for Social Sciences (SPSS) version 20.0 software for Windows.

## Results

Table 1 represents the descriptive analysis of the surgeons, in the EG group, 75.0% were men, in GS 66.7% and in CG 50.0%. Regarding the dominant hand, all participating surgeons from the three groups were right-handed. In the video games practice only 58.3% of the EG group and 66.7% of the SG play them for more than 3 h. In the TAPP technique, no surgeon in training demonstrated confidence in performing the technique.

**Table 1:** Sociodemographic characteristics, surgical and physical experience of the participants.

Variables			Surgeon group												P-value
			EG				SG				CG				
Age (years)			29.00 ± 2.73†				34.67 ± 1.53†				40.50 ± 6.36†				-
Sex															
	Male		9		75.0%		2		66.7%		1		50.0%		0.762
	Female		3		25.0%		1		33.3%		1		50.0%		
Dominant hand															
	Right-handed		12		100.0%		3		100.0%		2		100.0%		1.000
	Left-handed		0		0.0%		0		0.0%		0		0.0%		
Game (more than 3 h/week)															
	No		5		41.7%		1		33.3%		2		100.0%		0.270
	Yes		7		58.3%		2		66.7%		0		0.0%		
Ability with musical instrument															
	No		11		91.7%		1		33.3%		2		100.0%		0.047
	Yes		1		8.3%		2		66.7%		0		0.0%		
Specialty															
	General surgery		12		100.0%		1		100.0%		0		100.0%		0.001
	Others		0		0.0%		2		66.7%		2		100.0%		
Which laparoscopic procedures have confidence in performing															
	Cholecystectomy		5		41.7%		3		100.0%		2		100.0%		0.084
	Appendectomy		3		25.0%		3*		100.0%		2*		100.0%		0.019
	Hernioplasty		0		0.0%		0		0.0%		2*		100.0%		< 0.001
	Fundoplication		0		0.0%		1		33.3%		2*		100.0%		0.002
Residence time/performance			11.67 ± 7.69				64.00 ± 18.33*				150.00 ± 42.43* †				< 0.001
Estimated observation time/assistance with video surgery (hours)			192.50 ± 321.59				4253.33 ± 1397.33*				12000.00 ± 2828.43* †				< 0.001
Estimated time of previous training in simulators (hours)			15.83 ± 27.46				63.33 ± 40.41*				425.00 ± 106.07* †				< 0.001
Estimated time of previous operating room training (hours)			82.33 ± 137.60				600.00 ± 100.00*				4720.00 ± 1244.51* †				< 0.001
Number of TAPP hernias performed			0.08 ± 0.29				0.67 ± 1.15				390.00 ± 339.41* †				< 0.001
Laparoscopy experience time (months)			12.42 ± 8.71				6800 ± 13.86*				150.00 ± 42.43* †				< 0.001

* Friedman/Dunn Test,

† Kruskal-Wallis/Dunn Test (mean ± standard deviation).

Table 2 describes the relationship of the learning curve between the first session and the final session in the three groups. It was possible to observe that there was no significant difference among the groups, only within the EG group that were the surgeons in training. The evolution of the mean laparoscopic skills scores during the sessions can also be observed in Table 2. The skills evaluated during the execution of the technique were four: ambidexterity, stereotaxis, hapticity and central effect, each corresponding to one point. The surgeons in training presented in the first session an average of 1.08 ± 0.29 and in the sixth of 3.50 ± 0.90 (p = 0.001).

**Table 2:** Learning curve and procedure time of surgeons in training during training sessions.

				Training sessions				
				S1		S6		p-Value
1st stage		EG		1.25 ± 0.42aA		3.25 ± 0.62bB		0.002*
		SG		3.33 ± 0.57B		0.285†		
		CG		4.00 ± 0.00B				
P-value				< 0.001†				
2nd stage		EG		1.08 ± 0.67aA		2.58 ± 0.51cB		0.019*
		SG		2.58 ± 0.31B		0.263†		
		CG		3.00 ± 0.00B				
P-value				< 0.001†				
3rd stage		EG		1.00 ± 0.43aA		2.08 ± 0.29cB		0.004*
		SG		2.00 ± 0.00B		0.831†		
		CG		2.00 ± 0.00B				
P-value				0.001†				
4th stage		EG		1.08 ± 0.67aA		2.92 ± 0.29bB		< 0.001*
		SG		2.67 ± 0.58B		0.468†		
		CG		3.00 ± 0.00B				
P-value				0.001†				
5th stage		EG		0.91 ± 0.29ªA		1.91 ± 0.29bB		< 0.001*
		SG		2.00 ± 0.00B		0.831†		
		CG		2.00 ± 0.00B				
P-value				< 0.001†				
Laparoscopic skills		EG		1.08 ± 0.29aA		3.50 ± 0.90cC		< 0.001*
		SG		5.00 ± 0.00B		0.012†		
		CG		5.00 ± 0.00B				
P-value				< 0.001†				
Time (min)		EG		7.82 ± 3.74aA		4.73 ± 2.62aB		0.555*
		SG		4.74 ± 1.79B		0.918†		
		CG		3.98 ± 0.58B				
P-value				0.204†				
Score		EG		4.89 ± 0.92aA		14.22 ± 0.97dC		< 0.001*
		SG		15.00 ± 0.00B		0.039†		
		CG		16.00 ± 0.00B				
P-value				< 0.001†				

* Friedman / Dunn test,

† Kruskal–Wallis / Dunn test (mean ± standard deviation).

Different lowercase letters = significant difference between assessment moments, Different uppercase letters = Significant difference between groups.

## Discussion

Even though it is hardly used in regions with limited health resources, TAPP laparoscopic correction has recurrence rates equal to or lower than previous repairs associated with the benefit of low morbidity with rapid work return^15^. Transabdominal preperitoneal inguinal hernia repair rates range from 10 to 48%^16^, and almost half of surgeons had never performed laparoscopic repair, corroborated by this study, in which only two surgeons in training had performed / assisted a laparoscopic hernia^13^.

Participants in this study describe the need for TS in the first year of training. As in the study by Chipman *et al*.^17^ and Schmidt *et al*.^18^, who say that a good surgical curriculum training with the best technical training possible should be introduced as early as possible in the surgeon’s training. Among surgeons in training, 75% report that the frequency of the simulation should take place every two weeks. Trevisonno *et al*.^13^ state that both experienced and in-training surgeons should perform simulated training followed by supervised procedure only after it can be performed without external intervention.

Within EG group, 58% of participants reported that their previous gaming activities shortened their laparoscopic skills, which did not show statistical significance and the literature also has conflicting data when stating that video games shorten the curve. The biggest gain seems to be related to the psychomotor component^18^^–^ 21. It is known that the resting period is important for the retention of psychomotor skills as stated^22^.

The insertion of a systematized curriculum allows the training surgeon to easily and quickly identify the steps of the technique execution ensuring a greater confidence in performing the procedure with objectivity and safety for the patient. Systematizing simulation training enables the construction of performance evaluation tools allowing the technique validation for Cristancho *et al*.^23^.

In this study, the TS lasted six consecutive weeks, each session being short, with an average of 15 min. According to Mitchell *et al*.^24^, the ideal simulation program should consist of sessions that do not exceed one hour and have weekly intervals, as these conditions are associated with the keeping and improvement of newly acquired skills.

As going through the six sessions, the surgeon in training acquired the skills through of the curriculum systematic training, a fact already noticeable, with statistical difference from the third session that progresses further in thesixth session. When comparing the three groups after the sixthsession, the surgeon in training already has expertise comparable to the other groups, without statistical difference.

Regarding the time, although surgeons in training have a decreasing average time, it has no statistical difference when compared to each other in the first and last session and when compared to the sixth session with the other two groups. It can be inferred that perhaps the execution beyond six sessions in any given session will present statistical difference when comparing each other and between groups.

## Conclusions

The developed 3D model used low-cost material and was easy to reproduce, which allowed a systematization, facilitating the method of the TAPP laparoscopic repair technique through the use of the curriculum, allowing the training surgeon to gain upward laparoscopic skills equipping the surgeons with or without experience after the simulation sessions. The results comparison of the execution in each stage of the curriculum among the groups showed no significance, but in the first session of the EG group, to the last, it was possible to observe a significant improvement, demonstrating the effectiveness of the 3D model and the systematized curriculum developed.
